# Deep Learning Predicts Overall Survival of Patients With Unresectable Hepatocellular Carcinoma Treated by Transarterial Chemoembolization Plus Sorafenib

**DOI:** 10.3389/fonc.2020.593292

**Published:** 2020-09-30

**Authors:** Lei Zhang, Wei Xia, Zhi-Ping Yan, Jun-Hui Sun, Bin-Yan Zhong, Zhong-Heng Hou, Min-Jie Yang, Guan-Hui Zhou, Wan-Sheng Wang, Xing-Yu Zhao, Jun-Ming Jian, Peng Huang, Rui Zhang, Shen Zhang, Jia-Yi Zhang, Zhi Li, Xiao-Li Zhu, Xin Gao, Cai-Fang Ni

**Affiliations:** ^1^Department of Interventional Radiology, The First Affiliated Hospital of Soochow University, Suzhou, China; ^2^Suzhou Institute of Biomedical Engineering and Technology, Chinese Academy of Sciences, Suzhou, China; ^3^Department of Interventional Radiology, Zhongshan Hospital, Fudan University, Shanghai, China; ^4^Shanghai Institution of Medical Imaging, Shanghai, China; ^5^Hepatobiliary and Pancreatic Interventional Treatment Center, Division of Hepatobiliary and Pancreatic Surgery, The First Affiliated Hospital, Zhejiang University School of Medicine, Hangzhou, China

**Keywords:** hepatocellular carcinoma, transarterial chemoembolization, sorafenib, deep learning, biomarker

## Abstract

**Objectives:**

To develop and validate a deep learning-based overall survival (OS) prediction model in patients with hepatocellular carcinoma (HCC) treated with transarterial chemoembolization (TACE) plus sorafenib.

**Methods:**

This retrospective multicenter study consisted of 201 patients with treatment-naïve, unresectable HCC who were treated with TACE plus sorafenib. Data from 120 patients were used as the training set for model development. A deep learning signature was constructed using the deep image features from preoperative contrast-enhanced computed tomography images. An integrated nomogram was built using Cox regression by combining the deep learning signature and clinical features. The deep learning signature and nomograms were also externally validated in an independent validation set of 81 patients. C-index was used to evaluate the performance of OS prediction.

**Results:**

The median OS of the entire set was 19.2 months and no significant difference was found between the training and validation cohort (18.6 months vs. 19.5 months, *P* = 0.45). The deep learning signature achieved good prediction performance with a C-index of 0.717 in the training set and 0.714 in the validation set. The integrated nomogram showed significantly better prediction performance than the clinical nomogram in the training set (0.739 vs. 0.664, *P* = 0.002) and validation set (0.730 vs. 0.679, *P* = 0.023).

**Conclusion:**

The deep learning signature provided significant added value to clinical features in the development of an integrated nomogram which may act as a potential tool for individual prognosis prediction and identifying HCC patients who may benefit from the combination therapy of TACE plus sorafenib.

## Introduction

Almost 80% of patients with hepatocellular carcinoma (HCC) are initially diagnosed at the intermediate or advanced stage, hence being unqualified for curative treatments such as resection and ablation (1, 2). As demonstrated by two controlled randomized trials and the BRIDGE study, transarterial chemoembolization (TACE) is the most common therapeutic option for unresectable hepatocellular carcinoma (HCC), and is recommended for intermediate stage HCC (Barcelona Clinic Liver Cancer (BCLC) stage B) by most guidelines (3–7). However, the release of angiogenic factors such as vascular endothelial growth factor (VEGF) induced by TACE may increase the recurrence and progression rate of HCC (8, 9).

Sorafenib, a multikinase inhibitor, was the first oral molecular targeting agent to significantly improve overall survival (OS) and time-to-tumor progression (TTP) in patients with advanced HCC (10, 11). Theoretically, owing to acute hypoxia triggered by TACE which leads to the upregulation of VEGF, the combination of TACE and sorafenib may inhibit both revascularization and tumor proliferation (12, 13). Recently, the TACTIS trial clearly showed that TACE plus sorafenib significantly improved clinical outcomes in patients with unresectable HCC, which indicated that this combination therapy was effective and feasible in routine practice (14). However, several clinical trials failed to contribute compelling evidence for the combination of sorafenib and TACE, apart from the trial design which described the duration of sorafenib administration and TACE treatment regimen, the failure could be mainly due to the vast heterogeneity of unresectable HCCs, leading to differences in individual response (15–18). Therefore, a personalized prediction biomarker or model which can identify patients who may benefit from the combination therapy is crucial for treatment decision. Previous studies indicated that there was a potential link between adverse events (AEs) and favorable outcomes, which concluded that the earlier the AEs such as dermatological AEs and hypertension occurred, the longer the overall survival (OS) of patients on the combination therapy (19, 20). Nevertheless, biomarkers or models which provide accurate prognosis predictions are still lacking.

As a non-invasive examination tool used routinely in clinical practice, medical imaging can provide comprehensive evaluations of tumor heterogeneity, and previous studies found that image-based deep learning technologies showed promising capabilities in the development of accurate prediction models (21, 22). Specifically, the transfer learning strategy makes it possible to implement deep learning on relatively small datasets (22, 23). In this study, we conducted a multicenter study to establish and validate a deep learning-based prognosis prediction model for HCC patients treated with the combination of TACE and sorafenib.

## Materials and Methods

### Study Design

This retrospective multicenter study enrolled consecutive treatment-naïve HCC patients who were treated with the combination of TACE and sorafenib between 2011 and 2016. Data of patients from center A and B were used as the training set for the development of the prognosis prediction model, and data of patients from center C were used as the validation set for independent model test. The study was approved by the Institution Ethics Review Boards of the three mentioned centers. The need for informed consent was waived due to the retrospective nature of the study.

### Patients

The diagnosis of HCC was confirmed according to the European Association for the Study of the Liver (EASL) or the American Association for the Study of Liver Disease (AASLD) criteria (6, 7). The inclusion criteria were as follows: 1) patients were 18 years or older; 2) the Eastern Cooperative Oncology Group (ECOG) scores were 0 or 1; 3) patients with unresectable HCC which is clinically a heterogeneous group including those with inter-mediate and advanced stage (6); 4) Child-Pugh class A to B7; 5) adequate hematological, clotting, and renal function. Patients were excluded from the study if the following criteria were present: (1) absence of baseline imaging and clinical data; (2) comorbidity with other primary malignancies; (3) infiltrative HCCs with obscure borders; (4) contraindications to TACE or sorafenib treatment; (5) having received previous HCC-related treatment, including resection, ablation, TACE, and radiotherapy.

Relevant information was retrieved from the clinical database, including ECOG scores, Child Pugh class, number of tumors, tumor size, BCLC stage, hepatitis B virus (HBV) status, liver cirrhosis status, tumor distribution status, serum α-fetoprotein (AFP), alanine aminotransferase (ALT), aspartate aminotransferase (AST), and hepatitis B surface antigen (HBsAg) level. Continuous variables were transformed into categorical variables based on recognized cutoff values (24).

Preoperative contrast-enhanced computed tomography (CECT) scans were performed before treatment and both arterial and portal phases of CECT were obtained.

The details of CECT protocol was showed in the supplements.

### Treatment

Sorafenib (Bayer Healthcare, Leverkusen, Germany) was administrated orally to patients within 1 week after every session of TACE. To preserve liver function, sorafenib administration was stopped before the day of each TACE session. In principle, the dose of sorafenib was 400 mg twice daily (800 mg/day). Nevertheless, treatment interruptions and dose reductions (400 mg once-daily, 400 mg alternated days) were permitted for drug-related adverse events (AEs), which were graded per Common Terminology Criteria of Adverse Events (CTCAE) version 5.0. Patients were excluded if they did not adhere to the regimen.

TACE was performed based on “on demand” mode. No patients underwent TACE using drug-eluting beads. Before chemoembolization, a diagnostic angiograph was performed to ensure the main portal vein was unobstructed and to determine the anatomy of the tumor vessels and hepatic artery. With a super-selection of segment or subsegment, a 2.7 F microcatheter (Progreat, Terumo, United States) was advanced into the feeding vessels. As selected according to the practice of each center, chemoembolization was performed with intra-arterial doxorubicin (10–50 mg) and oxaliplatin (100–200 mg) mixed with lipiodol (2–20 ml, lipiodol ultra-fluid; Guerbet, France) followed by injection of gelfoam particles. The injection volume of the emulsion was determined based on the tumor volume. Before performing additional TACE sessions, good performance status was essential. Patients would receive the best supportive care if they were not candidates for further TACE sessions. All TACE procedures were performed by several interventional radiologists with more than 8 years of experience.

### Clinical Endpoints and Follow-Up

The primary endpoint of the study was OS and the prediction models, which were built based on it. OS was defined as the time from the initial TACE treatment of HCC until any cause of death. In surviving patients, the censoring date was defined as the last follow-up (September 30, 2019). The secondary endpoint was progression-free survival (PFS). PFS was defined as the time from the date of TACE until the time of radiological progression by the modified Response Evaluation Criteria in Solid Tumors (mRECIST). Radiological progression was assessed by two independent radiologists who were blinded to the clinical information. In patients without death or progression, the censoring date was defined as the last radiological assessment date. Patients received follow-up of CECT every 4 weeks after each TACE session and every 8–12 weeks after disease stability has been attained. Follow-up duration was measured from the day of diagnosis to last visit or death.

### Deep Learning Signature Building

**FIGURE 1:**
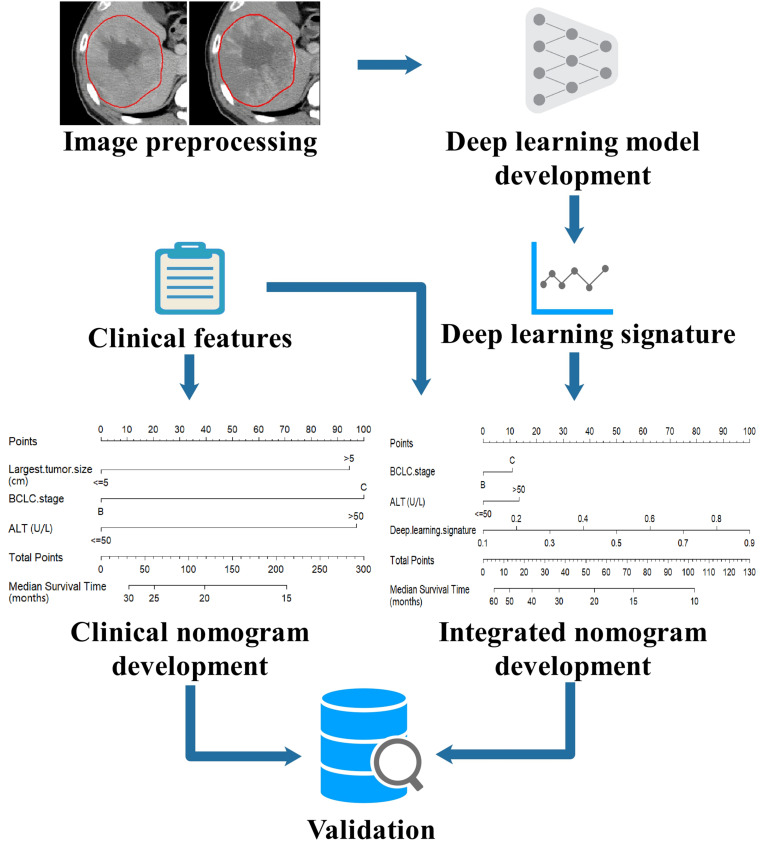
Workflow of modeling in this study. The CECT images were preprocessed by image registration, tumor delineation and image standardization, then the images were input into a deep learning model to build the deep learning signature. The deep learning signature and the clinical features were combined to develop an integrated nomogram. For comparison, a clinical nomogram was also built using only the clinical features. The nomograms were externally validated in an independent validation set.

The modeling workflow of this study is shown in Figure 1. The CECT images of the arterial and portal phases were aligned using open-source Insight Segmentation and Registration Toolkit (ITK, version 4.7.2^1^)(25). The tumor region of interest (ROI) was manually delineated in 2D slices of both AP and PVP using MITK software (version 2016.11.3^2^) by a radiologist with 10 years of experience, and then confirmed by a radiologist with 23 years of experience. The representative slices with the largest tumor ROI were selected, and square images with the size of 224 × 224 pixels whose center was located at the centroid of tumor ROI were generated. All images were processed by a z-score standardization, which consisted of subtracting the mean intensity and division by the standard deviation of intensity.

The deep learning model was adapted to decode the prognostic signal of tumors on CECT images. The training of deep learning models is computationally expensive and requires large number of images because of its millions of learnable parameters to estimate. To address the lack of data, a highly effective technique known as transfer learning was employed by leveraging large data set from computer vision domain (23). In this way, a deep learning model with DenseNet-121 architecture (26) was trained using ImageNet dataset^3^. DenseNet is a state-of-the-art convolutional neural network (CNN) which demonstrates significant improvements over traditional CNNs on highly competitive object recognition benchmark tasks, and it requires less computational cost and has fewer parameters which confers the model a smaller size and easier accessibility for application. ImageNet is a dataset for image classification which contains more than 14 million labeled natural images. The ImageNet dataset was used to train the DenseNet-121 model to derive model parameters, which conferred the general ability of image interpretation to the model, thus, the deep learning model can recognize the unique features of a specific category of images. In the DenseNet-121 model, the fully connected layer and softmax layer were removed, and the feature extraction module was used as deep image feature extractors. The DenseNet-121 model was used to extract 1024 deep image features from CECT images at each phase, respectively. The deep learning was implemented using Keras^4^ in Python with TensorFlow^5^ as the backend. The trained DenseNet-121 model is available online^6^. The technical details were described in the supplements.

An efficient two-stage modeling procedure was conducted to build the deep learning signature. In the first stage, the deep image features were ranked by mRMR, a multivariate ranking method (27). In the second stage, the top-ranked features were input into ElasticNet for the determination of feature weights and the building of the deep learning signature (28). 5-fold cross-validation was performed in modeling procedures to determine the optimal parameter configuration. The technical details were described in the supplements.

As the tumor ROI was manually delineated, the inter-observer and intra-observer correlation coefficient (ICC) were introduced to examine the reproducibility of deep image features in the deep learning signature. Two radiologists with 10 years of experience performed the same delineation of the tumor ROI for all patients: radiologist 1 delineated the ROI twice at different times and radiologist 2 carried out the delineation once. The deep image features were extracted after each delineation. The inter-observer and intra-observer ICC of deep image features were computed to determine the reproducibility of features, and the features with intra or inter-observer ICC above 0.75 were considered to have high reproducibility.

For an intuitive understanding of mechanisms of the deep learning signature, the strategy of class activation map (CAM) was used to generate heat maps which could give a coarse location of the image area relevant to unfavorable prognosis (29). The technical details of heat map generation were documented in the supplements.

### OS Prediction Nomogram Development

The Cox regression method was used to build the OS prediction nomogram. The clinical features and the deep learning signature were utilized as the candidate prognostic factors and tested by univariate Cox regression analysis to select the factors which were significantly correlated to OS. Then, the selected prognostic factors were used in multivariate Cox regression analysis to obtain an integrated nomogram by a stepwise feature selection algorithm based on the Akaike information criterion (AIC) (30). For comparison, a clinical nomogram was also built using only the clinical features.

### Validation and Statistical Analysis

The performance of models in predicting OS was evaluated by calculating the C-index (31). The deep learning signature and nomograms built on the training set were independently tested on the validation set. The calibration of nomograms was assessed by comparing observed and predicted survival using root mean square error (RMSE), where a lower RMSE reflects better agreement between observations and predictions (32).

In statistical tests, the Mann–Whitney *U* test was used for numerical variables, and Fisher’s exact test was used for categorical variables. All statistical tests were two-sided and *P* < 0.05 was used to indicate statistical significance.

## Results

### Baseline Characteristics

**FIGURE 2:**
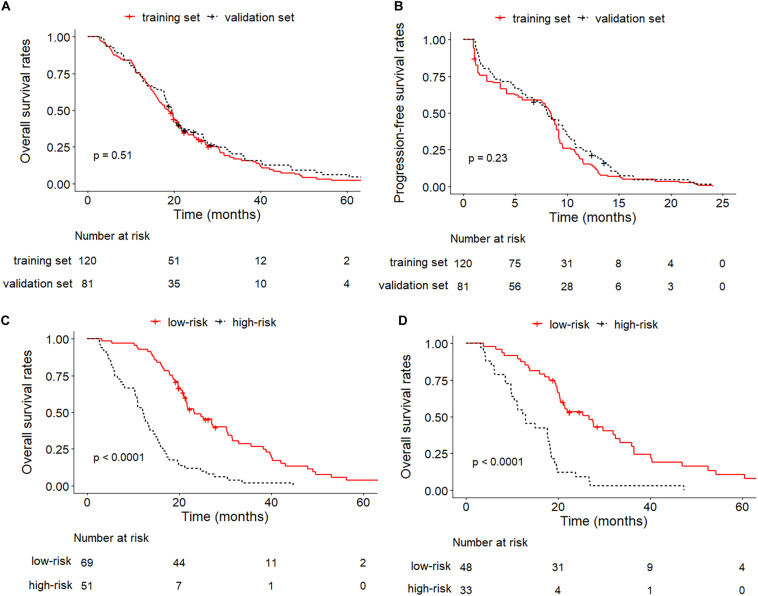
Kaplan–Meier curves between training and validation cohorts. **(A)** OS of training set and validation set; **(B)** PFS of training set and validation set; **(C)** OS of low-risk and high-risk groups in the training set; **(D)** OS of low-risk and high-risk groups in the validation set.

After enrollment, a total of 201 patients were included in this study (center A and B as the training set: *n* = 120, center C as the validation set: *n* = 81) (Supplementary Figure S1). The median OS and PFS of the entire set was 19.2 months (95% CI: 17.7–20.7) and 8.3 months (95% CI: 7.7–9.0) and no significant difference was found between the training and validation cohort (median OS: 18.6 (95% CI: 16.2–21.2) vs. 19.5 (95% CI: 17.8–21.9) months, *P* = 0.45; median PFS: 8.4 (95% CI: 7.5–9.0) vs. 8.1 (95% CI: 6.8–9.9) months, *P* = 0.23). The Kaplan–Meier curves of the training and validation sets for OS and PFS were plotted in Figures 2A,B. The detailed demographic characteristics of the enrolled patients in both sets were shown in Table 1.

**TABLE 1:** Baseline characteristics in the training and validation set.

Characteristic	Overall	Training set	Validation set	*P*
Gender				0.292
Male	175	107	68	
Female	26	13	13	
Age				0.475
≤55 years	108	67	41	
>55 years	93	53	40	
**Etiology**				
HBV	165	98	67	0.923
HCV	22	14	8	
Others	14	8	6	
Cirrhosis				0.384
Yes	115	72	43	
No	86	48	38	
Tumor distribution				0.359
Unilobar	136	78	58	
Bilobar	65	42	23	
Number of nodules				0.079
<3	83	56	27	
≥3	118	64	54	
Largest tumor size, median				0.374
≤5cm	76	42	34	
>5cm	125	78	47	
Portal vein invasion				0.146
Main portal vein	28	13	15	
First branch	57	30	27	
Second branch	7	5	2	
No	109	72	37	
Hepatic vein invasion				0.834
Yes	27	17	10	
No	174	103	71	
ECOG				0.157
0	180	104	76	
1	21	16	5	
Child-Pugh Class				0.442
A	184	108	76	
B	17	12	5	
BCLC stage				0.565
B	89	51	38	
C	112	69	43	
AST				0.313
≤40 U/L	90	50	40	
>40 U/L	111	70	41	
ALT				0.742
≤50 U/L	151	89	62	
>50 U/L	50	31	19	
AFP				0.229
≤400 ng/ml	71	38	33	
>400 ng/ml	130	82	48	
TACE sessions, median	2	2	2	0.579

*ECOG, Eastern Cooperative Oncology Group; BCLC, Barcelona Clinic Liver Cancer; HBV, hepatitis B virus; HCV, hepatitis C virus; AFP, α-fetoprotein; ALT, alanine aminotransferase; AST, aspartate aminotransferase; TACE, transarterial chemoembolization.*

The duration of sorafenib administration was 12.8 months (range: 1.2–45.4 months). The dose reductions and interruptions in 152 (75.6%) patients were mainly due to disease progression and AEs. No combination therapy-related deaths occurred during the follow-up. The AEs of patients were listed in Supplementary Table S1 of the supplements.

### Deep Learning Signature Building and Validation

There were 10 deep image features in the deep learning signature including 5 features extracted from arterial phase CECT and 5 features extracted from portal phase CECT, the names of the features and corresponding weights were detailed in Supplementary Table S2 of the supplements. All deep image features in the deep learning signature had ICC above 0.75. The deep learning signature achieved a C-index of 0.717 (95% CI: 0.709–0.726) in the training set, and it was validated to have good prediction performance with a C-index of 0.714 (95% CI: 0.702–0.727) in the validation set.

**FIGURE 3:**
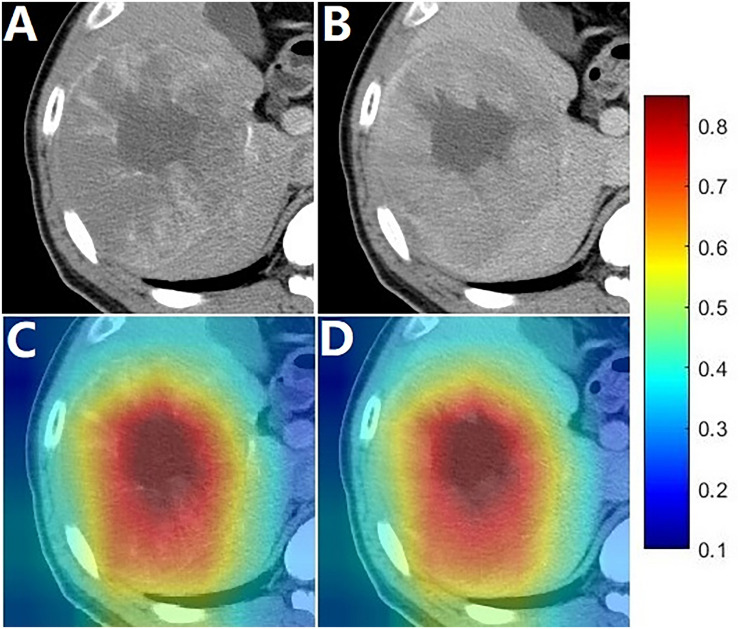
Images of a patient with an OS of 13.6 months. **(A,B)** were arterial phase and portal phase CECT images, respectively; **(C,D)** shows the heat map superimposed on the arterial phase and portal phase CECT images.

Heat maps were generated to provide a coarse location of the tumor region relevant to unfavorable prognosis. Figure 3 illustrates an example of CECT images with superimposed heat maps, where the areas in deeper red indicated a stronger correlation with unfavorable prognosis. The core area colored deepest red was located in the hypodense mass, and the general red area covered almost the entire tumor.

### OS Prediction Nomogram Development and Validation

**FIGURE 4:**
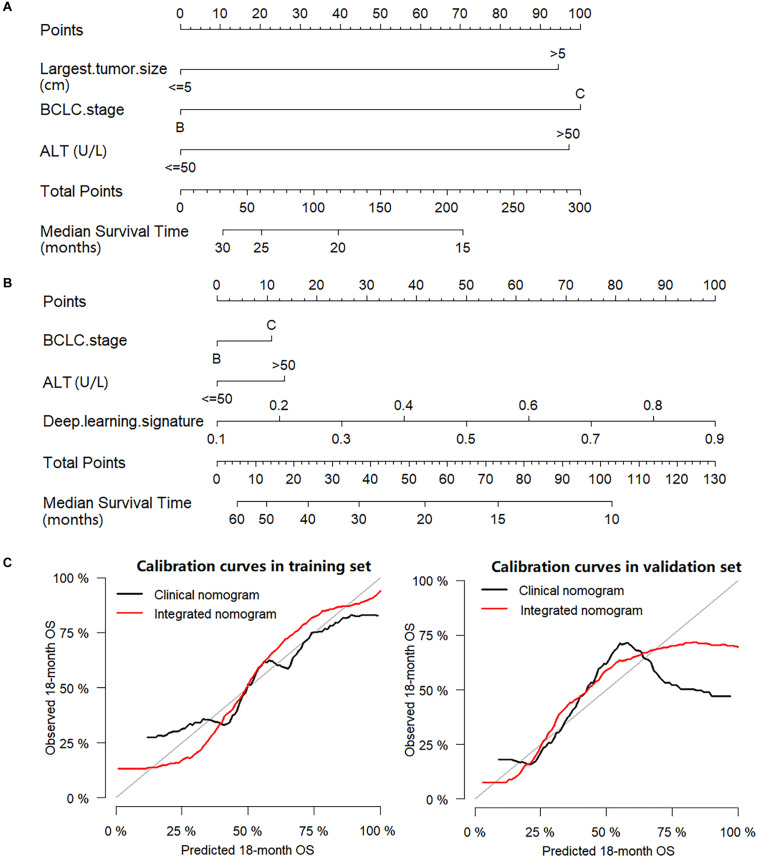
Nomograms and calibration curves. **(A)** Clinical nomogram; **(B)** Integrated nomogram; **(C)** Calibration curves of nomograms in the training set and validation set.

The BCLC stage, largest tumor size, AFP, ALT and deep learning signature were identified as prognostic factors correlated to OS in the univariate analysis (Supplementary Table S3). When only the prognostic clinical features were used in multivariate Cox regression analysis, BCLC stage, largest tumor size and ALT were identified as independent prognostic factors (Table 2) and a clinical nomogram was built (Figure 4A). By including all prognostic factors in the multivariate Cox regression analysis, the largest tumor size failed to remain as an independent prognostic ability, while BCLC stage, ALT and deep learning signature were identified as independent prognostic factors (Table 2), and an integrated nomogram was built using these factors (Figure 4B).

**TABLE 2:** Nomograms built using multivariate Cox regression analysis.

Characteristic	Clinical nomogram		Integrated nomogram	
		
	HR (95% CI)	*P*	HR (95% CI)	*P*
BCLC stage (C vs. B)	1.968 (1.307–2.964)	0.001	1.540 (1.016–2.334)	0.041
Largest tumor size (>5 vs. ≤5)	1.896 (1.222–2.949)	0.004	–	–
ALT (>50 vs. ≤50)	1.931 (1.245–2.993)	0.003	1.703 (1.099–2.639)	0.017
Deep learning signature (0.6 vs. 0.4)	–	–	2.688 (1.970–3.668)	<0.001

*HR, Hazard Ratio; CI, Confidence Interval.*

In the training set, the C-index for the integrated nomogram (0.739, 95% CI: 0.731–0.748) for the prediction of OS was significantly higher than that of the clinical nomogram (0.664, 95% CI: 0.654–0.673, *P* = 0.002). Consistent results were found in the validation set, where the C-index remained significantly greater for the integrated nomogram (0.730, 95% CI: 0.717–0.742) compared with the clinical nomogram (0.679, 95% CI: 0.667–0.691, *P* = 0.023). The calibration plots of nomograms were plotted in Figure 4C. In the training set, the RMSE was 0.068 for the clinical nomogram and 0.062 for the integrated nomogram. In the validation set, the RMSE of the clinical nomogram was 0.192 and the RMSE of the integrated nomogram was 0.105.

### Performance of Integrated Nomogram in Stratifying Risk of Patients

The median value of the scores predicted by the integrated nomogram was determined as the cutoff in stratifying the patients in the training cohort into two subgroups, where the subgroup with scores higher than median score were classified as the high-risk group, and the other subgroup was classified as the low-risk group, and the patients in the low-risk group achieved better OS than the high-risk group (*P* < 0.001). After applying the same cutoff value in stratifying patients in the validation set, stratification into high and low-risk subgroups also achieved significantly distinct OS (*P* < 0.001). The Kaplan–Meier curves of low-risk and high-risk groups in the training and validation sets were illustrated in Figures 2C,D.

## Discussion

A multicenter study was conducted to develop and validate an OS prediction model for HCC patients treated with a combination of TACE and sorafenib, where an integrated nomogram which was built by incorporating a deep learning signature and clinical features showed significant improvement compared to the clinical nomogram. When comparing OS of this study to that of other trials, OS of the SPACE trial did not reach the median value, the TACTIS trial did not analyze OS, and the target population of the STAH and post-TACE trial was advanced stage HCCs and unresectable HCCs with a response after TACE (14, 15, 33, 34). The median OS of this study was consistent with the TACE-2 trial (median OS, 18.8 months), which suggested that the cohort in the present study is representative of real-world patients receiving TACE plus sorafenib for unresectable HCC (16).

Several clinical trials which attempted to address the improvement in OS of the combination treatment of TACE plus sorafenib in HCC patients have ended in failure (16, 33, 34). The failure of the negative trials could mainly be due to the deficiency of effective biomarkers (18). For HCC patients, it is known that baseline α-fetoprotein concentration and other biomarkers such as miR-26 miRNA precursor, epithelial cell adhesion molecule are suggested to be correlated with the outcomes (35, 36). In addition, more than 40 gene signatures have been described in terms of molecular-guided prognosis prediction (37). Nevertheless, none of them have yet to become a tangible tool in clinical practice mainly due to the impact of intra- and inter-tumor heterogeneity (38, 39). Another reason may be that the molecular biomarkers were identified by the specimens resected from patients at earlier stages but have not proven to be predictors of a response to systematic therapies such as sorafenib (37). A few studies showed that early-onset sorafenib-related AEs may be potential biomarkers for patients undergoing treatment with sorafenib (20, 40). Recently, the onset of hypertension and sorafenib-related dermatological AEs were demonstrated to be early biomarkers in patients with HCC who were treated with TACE plus sorafenib (19). Nevertheless, on the basis of complexity of the histopathological and biological heterogeneity of HCC, the multi-target treatment mechanisms of sorafenib in addition to the factors mentioned above, these biomarkers are unable to strongly predict the outcomes of patients with HCC who were treated with TACE plus sorafenib (41).

The image-based deep learning technology enabled the development of powerful prognosis biomarkers to predict outcomes in malignant tumors such as lung cancer (42), nasopharyngeal carcinoma (43) and gliomas (44). With the transfer learning strategy, the deep learning model was employed to build the deep learning signature in this relatively small data set. The entire modeling procedure was efficient and easy to implement with open-source programs. As shown in the results, the deep learning signature was highly correlated to OS. In the heat map, it was indicated that the deep learning signature could capture local features, where the deepest red areas identified were associated with the hypodense mass which may refer to necrosis (Figure 3). The arterial flow may decrease due to larger tumor growth, further dedifferentiation and progression to poorly differentiated HCC (45). Moreover, in very advanced HCCs, compression closure of tumor capillaries and the diminishing of newly developed blood vessels occurred due to the increasing interstitial pressure caused by rapid cell proliferation in the tumor center (46). Given these factors, necrosis may emerge in HCCs, which might make it a predictor of prognosis. In the heat map, the general red area almost covered the entire tumor, which suggested that the deep learning signature could capture global features including the tumor size, which is a predictor of poor prognosis in HCC (47, 48).

As presented in the study, a clinical nomogram was built, which included the BCLC stage, largest tumor size and ALT to predict individual outcomes. The BCLC stage relies on a composite of tumor burden, degree of liver damage, and cancer-related symptoms, providing a useful framework for clinical practice (6). Numerous studies have shown that larger tumor sizes are predictors of poor prognosis in HCC (47, 48). Moreover, ALT which was often utilized in the evaluation of liver function in clinical practice, was demonstrated to be linked with survival in patients with HCC (49). These were also true in our cohort where larger tumor sizes, higher ALT levels and BCLC C stage were associated with poor OS. An integrated nomogram was built by incorporating clinical features and a deep learning signature, where the integrated nomogram achieved a higher C-index and lower RMSE than that of the clinical nomogram, which indicated that the deep learning signature provided significant added value to clinical features. The reason may be because the deep learning signature could make predictions by capturing both the global and local features of tumors, and it comprehensively reflected on the tumor size and heterogeneity which were established prognostic factors (39, 50). This may also explain the exclusion of largest tumor size as a prognostic factor, as the deep learning signature has already contained the information of tumor size which belongs to the tumor global feature, which was consistent with the demonstration in the heat map.

Some limitations of this study should be acknowledged. First, because of its retrospective nature, selection bias may have existed and the cohort may not represent the entire population of patients with unresectable HCC. Nevertheless, there were no significant differences in the baseline characteristics between the three centers. Second, in this study, sequential administration rather than concurrent administration of the combination treatment may limit the efficacy of treatment. However, physicians preferred the sequential approach to avoid possible AEs in clinical practice. Third, because of the limited data, the study population included BCLC C stage HCC, where TACE is not routinely recommended. Hence, further study of BCLC B stage population is warranted. Last but not least, the entire modeling procedure was not fully automatic, and tumor delineation was required to reduce the image size and to eliminate background noise which ensured that the deep learning model could focus on the signal of the tumor. In the future, it is hoped that an end-to-end deep learning model can be trained on a large scale of dataset without the need for pre-processing procedures.

## Conclusion

In conclusion, the current study demonstrated that the CECT-based deep learning signature could be used as a novel biomarker for OS prediction in patients with HCC undergoing TACE plus sorafenib treatment. Additionally, we built an integrated nomogram combining the clinical features and the deep learning signature to further improve the prediction of OS which could thereby act as a potential tool for the development of individual treatment strategies and identifying potential patients with HCC who may benefit from such a combination therapy.

## Data Availability Statement

The raw data supporting the conclusions of this article will be made available by the authors, without undue reservation.

## Ethics Statement

The studies involving human participants were reviewed and approved by The First Affiliated Hospital of Soochow University. The ethics committee waived the requirement of written informed consent for participation. Written informed consent was obtained from the individual(s) for the publication of any potentially identifiable images or data included in this article.

## Author Contributions

All authors contributed to review and critical revision of the manuscript and approved the final version of the manuscript. XG, C-FN, LZ, and WX contributed to the study concept and design. LZ, B-YZ, Z-HH, PH, SZ, M-JY, G-HZ, W-SW, ZL, and X-LZ contributed to acquisition of data. LZ, WX, X-YZ, and J-MJ contributed to analysis and interpretation of data. WX, RZ, and J-YZ contributed to statistical analysis. LZ and WX contributed to drafting of the manuscript. The corresponding author had full access to all of the data and took full responsibility for the veracity of the data and the statistical analyses.

## Conflict of Interest

The authors declare that the research was conducted in the absence of any commercial or financial relationships that could be construed as a potential conflict of interest.

## Footnotes

1 https://itk.org/

2 http://www.mitk.org/

3 https://www.image-net.org

4 https://github.com/keras-team/keras/

5 https://www.tensorflow.org/

6 https://github.com/flyyufelix/DenseNet-Keras

## Abbreviations

95% CI: 95% confidence interval
AASLD: American Association for the Study of Liver Diseases
AEs: adverse events
AFP: α -fetoprotein
AIC: Akaike information criterion
ALT: alanine aminotransferase
AST: aspartate aminotransferase
BCLC: Barcelona Clinic Liver Cancer
CAM: class activation map
CECT: contrast-enhanced computed tomography
C-index: concordance index
CT: computed tomography
CTCAE: Common Terminology Criteria of Adverse Events
EASL: European Association for the Study of the Liver
ECOG: Eastern Cooperative Oncology Group
GAP: global average pooling
HBsAg: Hepatitis B virus surface antigen
HBV: Hepatitis B virus
HCC: hepatocellular carcinoma
IRBs: Institutional Review Boards
mRECIST: modified Response Evaluation Criteria in Solid Tumors
MRI: magnetic resonance imaging
OS: overall survival
PFS: progression-free survival
RMSE: root mean square error
ROI: region of interest
SAEs: Severe Adverse Events
SD: standard deviation
TACE: transarterial chemoembolization
TTP: time-to-tumor progression
VEGF: vascular endothelial growth factor.
