# Supplementary material for: Deep Learning Predicts Overall Survival of Patients With Unresectable Hepatocellular Carcinoma Treated by Transarterial Chemoembolization Plus Sorafenib
Source: Front Oncol. 2020 Sep 30;10:593292. doi: 10.3389/fonc.2020.593292 (PMC7556271; doi:10.3389/fonc.2020.593292)
Supplement: Supplementary file 1 [file Data_Sheet_1.DOCX]

Supplement A: Contrast-enhanced computed tomography (CECT) protocol

CECT scans were performed with the Siemens SOMATOM Sensation 64 CT scanner (Erlangen, Germany) in three centers approximately 1 week before TACE treatment. The scanner was the same in the three centers. The parameters associated with the CT scan were as follows: 120 kV, 180 mAs; collimation, 2.5-5 mm; section thickness, 3-5 mm; reconstruction, 1.5 mm. For the triphasic protocol, images were obtained at 30-35, 60-70, and 180-300 seconds after the start of intravenous injection of non-ionic contrast medium (Ioversol; Optiray 240, Mallinckrodt, St. Louis, MO) at a dose of 60-110 mL (1.5 mL per kilogram of body weight) and at a rate of 2 to 3 mL/second.

Supplement B: Multicenter patient enrollment


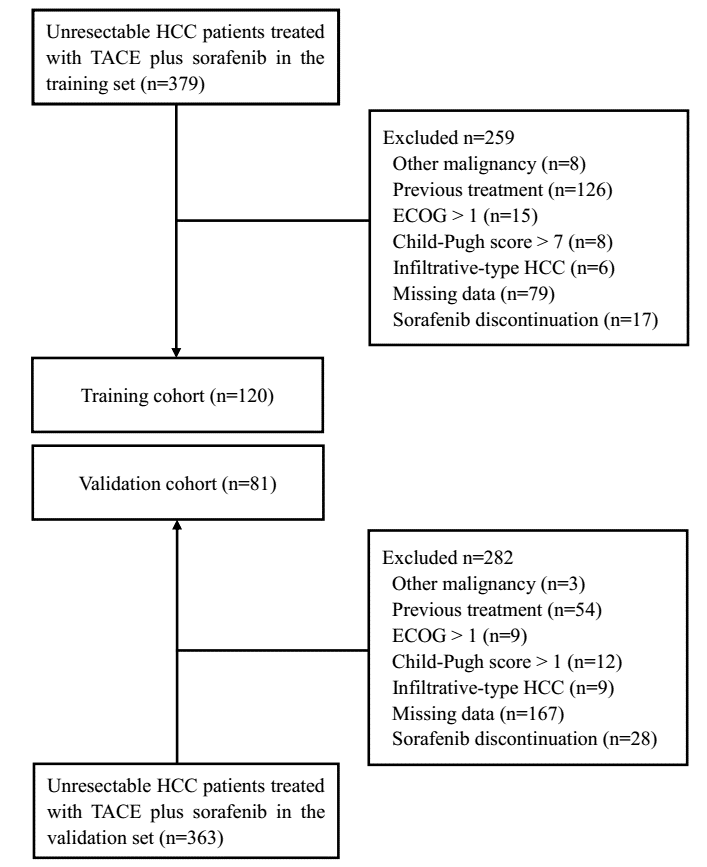


Figure S1. Flow chart of the multicenter patient enrollment.

Supplement C: The schematic of building the deep learning signature


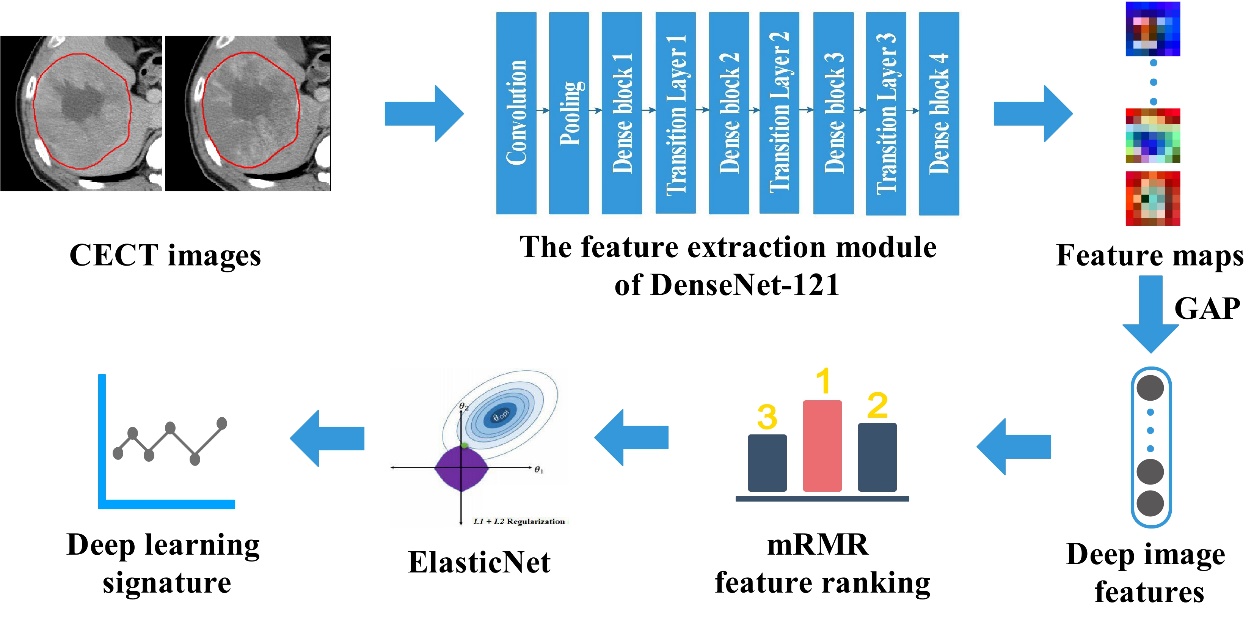


Figure S2. The schematic of building the deep image signature. The CECT images were input into the feature extraction module of DenseNet-121 to generate feature maps, then the feature maps were converted to deep image features by global average pooling (GAP). Useful deep image features were identified by the mRMR feature ranking method and input into ElasticNet to build the deep learning signature.

Supplement D: DenseNet and transfer learning

DenseNet is a state-of-the-art convolutional neural network (CNN) which demonstrates significant improvements over traditional CNNs on highly competitive object recognition benchmark tasks, and it requires less computational cost and has fewer parameters which confers the model a smaller size and easier accessibility for application[^1^](#_ENREF_1).

An advantage of DenseNet is the benefit from the design of dense block in its feature extraction module (Figure S3)[^1^](#_ENREF_1). The dense block introduced a dense connectivity, which is direct connections from any layer to all subsequent layers. For each layer, the feature-maps of all preceding layers are used as inputs, and its own feature-maps are used as inputs into all subsequent layers. Consequently, they alleviate the vanishing-gradient problem, strengthen feature propagation, encourage feature reuse, and substantially reduce the number of parameters[^1^](#_ENREF_1). The DenseNet-121 model was employed in this study[^1^](#_ENREF_1).

Since the DenseNet-121 model has a huge number of parameters for training and requires a large dataset, one method of addressing the lack of data in a given domain is to leverage data from a similar domain, a technique known as transfer learning^2^. ImageNet (https://www.image-net.org) is a dataset for image classification which contains more than 14 million labeled natural images. The ImageNet dataset was used to train the DenseNet-121 model to derive model parameters, which conferred the general ability of image interpretation to the model. The deep learning model was implemented using Keras (https://github.com/keras-team/keras/) in Python with TensorFlow (https://www.tensorflow.org/) as the backend. The trained DenseNet-121 model is available online (https://github.com/flyyufelix/DenseNet-Keras).

In the DenseNet-121 model, the fully connected layer and softmax layer were removed, and the feature extraction module was used as deep image feature extractors. The representative slices with the largest tumor ROI were selected, and square images with the size of 224×224 pixels whose center was located at the centroid of tumor ROI were generated, these images were masked by tumor ROI and taken as the input of the deep learning model. In the end, 1024 feature maps were extracted from arterial and portal phase CECT images by the feature extraction module respectively, and 2048 feature maps were obtained. These feature maps were transformed into 2048 deep image features by the global average pooling (GAP)[^1^](#_ENREF_1).

Figure S3. The feature extraction module of DenseNet-121. (A) The architecture of feature extraction module. (B) The schematic of dense block.


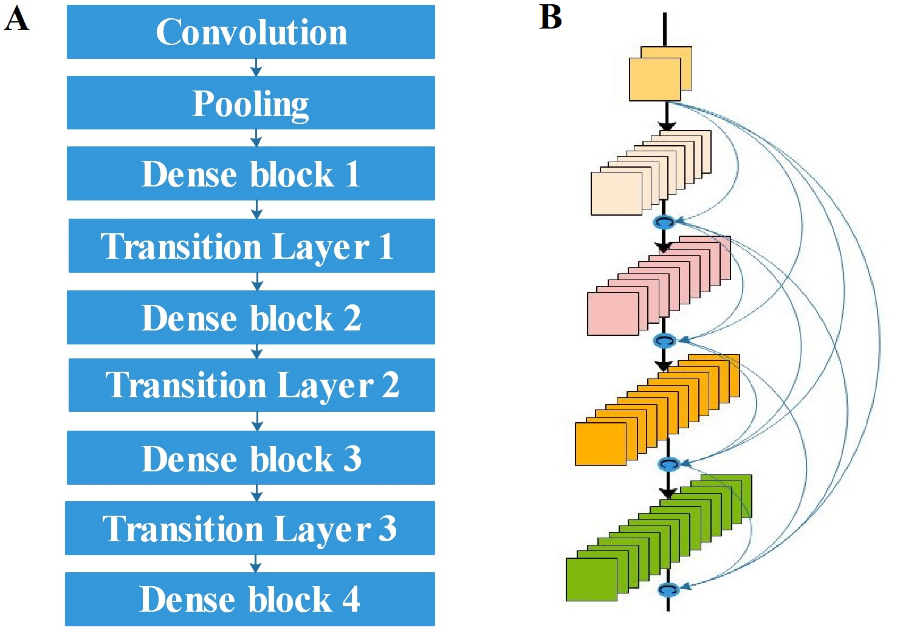


Supplement E: mRMR feature ranking algorithm

The feature ranking methods were the classifier-independent filter methods based on the heuristic scoring criterion, which allowed us to sort and select good features according to their scores. These methods are efficient, generalizable and scalable. This study used the Minimum Redundancy and Maximum Relevance (mRMR) method to select the most predictive deep image features[^3^](#_ENREF_3).

The mRMR is a feature ranking method that attempts to evaluate features by maximizing the relevance between the features and labels while simultaneously minimizing redundancy in the selected features. The mRMR scoring criterion is defined as the relevancy-redundancy tradeoff:

$$\emptyset=I\left( x_{i};c \right)-\frac{1}{\left| S \right|}\sum_{x_{i}\in S} I(x_{i};x_{j})$$

where *I* is the mutual information, *x* is the feature, *S* is the set of selected features, and *c* is the label. The first term $I(x_{i};c)$ describes the relevance between the feature and label, and the second term is the feature redundancy in the set of selected features. For the convenience of modeling, patients with OS values that were lower than the median OS were assigned as unfavorable prognosis, and the remaining patients were assigned as favorable prognosis. In this study, the top-ranked features were those highly relevant to the label (favorable or unfavorable prognosis) and low redundancy with *S*. The number of top-ranked features number $N$ is tuned from 5 to 50 with increments of 5. The mRMR was implemented using R programming language (https://www.r-project.org/, version 3.4.2).

Supplement F: ElasticNet

It has been demonstrated that the mRMR method could generate a compact feature set for subsequent modeling algorithms[^3^](#_ENREF_3). ElasticNet is an efficient general linear model (GLM) which combines the $L_{1}$ and $L_{2}$ penalties for regularization to implement an inherent feature weighting mechanism, and the result can be written as the linear weighted sum of features which is easy for interpretation[^4^](#_ENREF_4). There is a hyper-parameter α which is the weight of penalty need to be tuned in ElasticNet. ElasticNet was implemented by the R package “glmnet” with tuning α varied from 0.1 to 1 with increments of 0.1.

The weighted sum of the deep image features with the weights derived from ElasticNet was calculated as deep image score $S$ in equation (1), and the deep learning signature was built by transforming the deep image score $S$ to the probability of unfavorable prognosis $P$ using the sigmoid function in equation (2):

$S=w_{1}x_{1}+w_{2}x_{2}+\cdots+w_{i}x_{i}+b$ (1)

$P=1/{(1+exp(-S)})$ (2)

where $x_{i}$ is the deep image features, $w_{i}$ is the corresponding weights, and $\exp$ is the exponential function.

The ElasticNet was implemented using R programming language (https://www.r-project.org/, version 3.4.2).

Supplement G: Generation of heat map

It was demonstrated that the deep image features transformed by the GAP contains the localization information, and the importance of the image regions can be identified by projecting the weights of the output layer onto the feature maps[^5^](#_ENREF_5). A combined feature map was generated by the weighted sum of the feature maps which derived feature $x_{i}$ with the weights $w_{i}$ determined by ElasticNet, subsequently a heat map was generated by up-sampling the combined feature map to the same size of the input images to give a coarse location of the discriminative area. As the deep learning signature was designed to give the probability of unfavorable prognosis, a higher value in the heat map indicated a stronger correlation to unfavorable prognosis.

Supplement H: Adverse events of patients

Table S1. Adverse events of the training and validation cohort.

| Adverse events | Overall | Training cohort | Validation cohort | *P* |
| --- | --- | --- | --- | --- |
| Hand foot skin reaction |  |  |  | 0.774 |
| Yes | 97 (48.3) | 61 (50.8) | 39 (48.1) |  |
| No | 104 (51.7) | 59 (49.2) | 42 (51.9) |  |
| Bleed |  |  |  | 0.649 |
| Yes | 4 (2.0) | 3 (2.5) | 1 (1.2) |  |
| No | 197 (98.0) | 117 (97.5) | 80 (98.8) |  |
| Hypertension |  |  |  | 0.883 |
| Yes | 79 (39.3) | 48 (40.0) | 31 (38.3) |  |
| No | 122 (60.7) | 72 (60.0) | 50 (61.7) |  |
| Diarrhea |  |  |  | 0.110 |
| Yes | 85 (42.3) | 45 (37.5) | 40 (49.4) |  |
| No | 116 (57.7) | 75 (62.5) | 41 (50.6) |  |
| Rash |  |  |  | 0.268 |
| Yes | 37 (18.4) | 19 (15.8) | 18 (22.2) |  |
| No | 165 (81.6) | 102 (84.2) | 63 (77.8) |  |
| Abdominal pain |  |  |  | 0.473 |
| Yes | 106 (52.7) | 66 (55.0) | 40 (49.4) |  |
| No | 95 (47.3) | 54 (45.0) | 41 (50.6) |  |
| Fatigue |  |  |  | 0.141 |
| Yes | 51 (25.4) | 35 (29.2) | 16 (19.8) |  |
| No | 150 (74.6) | 85 (70.8) | 65 (80.2) |  |
| Nausea |  |  |  | 0.305 |
| Yes | 45 (22.4) | 30 (25.0) | 15 (18.5) |  |
| No | 156 (77.6) | 90 (75.0) | 66 (81.5) |  |
| Vomiting |  |  |  | 1.000 |
| Yes | 17 (8.5) | 10 (8.3) | 7 (8.6) |  |
| No | 184 (91.5) | 110 (91.7) | 74 (91.4) |  |
| Fever |  |  |  | 0.471 |
| Yes | 94 (46.8) | 59 (49.2) | 35 (43.2) |  |
| No | 107 (53.2) | 61 (50.8) | 46 (56.8) |  |
| Infection |  |  |  | 0.819 |
| Yes | 22 (10.9) | 14 (11.7) | 8 (9.9) |  |
| No | 179 (89.1) | 106 (88.3) | 73 (90.1) |  |

Data are number of patients, with percentage in parentheses.

Supplement I: Details of deep learning signature

Table S2. The deep image features and corresponding weights in the deep learning signature.

| Feature | Weight |
| --- | --- |
| P-100 | -0.1230 |
| P-191 | 0.1325 |
| P-489 | -0.1202 |
| P-755 | 0.1744 |
| P-981 | -0.1092 |
| A-598 | -0.1876 |
| A-745 | 0.1382 |
| A-914 | -0.1526 |
| A-978 | 0.1949 |
| A-1020 | 0.1570 |

Abbreviations: A or P in the feature name represents the arterial or portal phase of CECT, respectively. The number in the feature name indicates the identifier of feature map. For example, the P-100 feature was obtained from the GAP of the 100^th^ feature map extracted from the portal phase of CECT.

Supplement J: Univariate Cox regression analysis

Table S3. Results of univariate Cox regression analysis.

| Characteristic | HR (95% CI) | *P* |
| --- | --- | --- |
| Gender (female vs. male) | 0.805 (0.439-1.474) | 0.481 |
| Age (>55 vs. ≤55) | 0.864 (0.590-1.264) | 0.452 |
| ECOG (1 vs. 0) | 1.594 (0.916-2.772) | 0.095 |
| Child-Pugh class (B vs. A) | 1.029 (0.549-1.929) | 0.928 |
| BCLC stage (C vs. B) | 1.921 (1.300-2.840) | <0.001 |
| Tumor distribution (Bilobar vs. Unilobar) | 1.241 (0.839-1.835) | 0.278 |
| Number of nodules (<3 vs. ≥3) | 0.781 (0.532-1.147) | 0.207 |
| Largest tumor size (>5 vs. ≤5) | 2.080 (1.356-3.191) | <0.001 |
| Cirrhosis (0 vs. 1) | 0.836 (0.569-1.226) | 0.359 |
| HBsAg (0 vs. 1) | 0.864 (0.529-1.413) | 0.562 |
| AFP (≤400 vs. >400) | 0.619 (0.408-0.937) | 0.022 |
| ALT (>50 vs. ≤50) | 1.561 (1.023-2.381) | 0.037 |
| AST (≤40 vs. >40) | 0.683 (0.463-1.008) | 0.053 |
| Deep image signature (0.6 vs. 0.4) | 2.892 (2.143-3.901) | <0.001 |

Abbreviations: *HR, Hazard Ratio; CI, Confidence Interval*.

**Reference**

1. Huang G, Liu Z, van der Maaten L, Weinberger KQ. Densely Connected Convolutional Networks. *30th Ieee Conference on Computer Vision and Pattern Recognition (Cvpr 2017)* 2017: 2261-9.

2. Kermany DS, Goldbaum M, Cai WJ, et al. Identifying Medical Diagnoses and Treatable Diseases by Image-Based Deep Learning. *Cell* 2018; 172(5): 1122-+.

3. Peng HC, Long FH, Ding C. Feature selection based on mutual information: Criteria of max-dependency, max-relevance, and min-redundancy. *Ieee T Pattern Anal* 2005; 27(8): 1226-38.

4. Zou H, Hastie T. Regularization and variable selection via the elastic net (vol B 67, pg 301, 2005). *J R Stat Soc B* 2005; 67: 768-.

5. Zhou B, Khosla A, Lapedriza A, Oliva A, Torralba A. Learning Deep Features for Discriminative Localization. *Proc Cvpr Ieee* 2016: 2921-9.
